# Immune-Mediated Glycocalyx Remodeling in Hospitalized COVID-19 Patients

**DOI:** 10.1007/s10557-021-07288-7

**Published:** 2021-11-18

**Authors:** Sascha N. Goonewardena, Olga G. Grushko, Joanna Wells, Lauren Herty, Robert S. Rosenson, Jacob M. Haus, Scott L. Hummel

**Affiliations:** 1 Division of Cardiovascular Medicine, Department of Internal Medicine, University of Michigan, Ann Arbor, MI USA; 2 Ann Arbor Veterans Affairs Health System, Ann Arbor, MI USA; 3 Metabolism and Lipids Unit, Mount Sinai Heart, Icahn School of Medicine at Mount Sinai, New York, NY USA; 4 School of Kinesiology, University of Michigan, Ann Arbor, MI USA; 5 University of Michigan Frankel Cardiovascular Center, 1500 East Medical Center Drive, SPC 5853, Ann Arbor, MI 48109-5853 USA

**Keywords:** COVID-19, Inflammation, Vascular dysfunction, Glycocalyx

## Abstract

**Purpose:**

Vascular and immune dysfunction are hallmarks of severe acute respiratory syndrome coronavirus-2 (SARS-CoV-2) infections and coronavirus disease 2019 (COVID-19). Although our understanding of the pathogenesis of COVID-19 has rapidly evolved, much of the focus has been on the immune mechanisms underlying COVID-19. In addition to immune dysfunction, vascular injury is also associated with COVID-19 and is a major driver of clinical deterioration in SARS-CoV-2 infections. The glycocalyx (GAC), a sugar-based shell that surrounds all mammalian cells, is an important regulator of vascular and immune responses. In sepsis, vascular dysfunction contributes to acute respiratory distress syndrome (ARDS) by altering vessel integrity, promoting thrombosis, and accelerating inflammation, all of which are also present in COVID-19. Observational studies in sepsis have found an association between levels of circulating GAC degradation products with both organ dysfunction and mortality. Although vascular dysfunction is a hallmark of COVID-19, it remains unclear whether GAC disruption occurs in COVID-19 and if GAC disruption contributes to the clinical progression of COVID-19.

**Methods:**

In this prospective cohort study, we measured the GAC components syndecan-1 (SDC1) and hyaluronan (Hyal) along with inflammatory cytokines in 12 hospitalized COVID-19 patients and 8 healthy controls (HC).

**Results:**

In agreement with other studies, we found that inflammatory cytokines are elevated in hospitalized COVID-19 patients compared with HC [median (IQR), all units picograms per milliliter: IL-6 4.65 (3.32–9.16) vs 0.69 (0.55–0.89), *p* < 0.001; TNFα 4.49 (1.87–8.03) vs 0.04 (0.04–0.84), *p* < 0.001]. Additionally, we found that the GAC components SDC1 and Hyal are also elevated in COVID-19 patients [median (IQR), all units picograms per milliliter: SDC1: 247.37 (101.43–458.26) vs 84.8 (52.88–123.59), *p* = 0.036; Hyal: 26.41 (16.4–35.1) vs 3.01 (1.66–4.61), *p* < 0.001].

**Conclusion:**

We propose that GAC markers offer insights into the pathobiology of COVID-19, potentially guide therapeutic approaches, and could aid in early risk stratification that is particularly beneficial in phasic diseases such as COVID-19.

## Introduction

Coronavirus disease 2019 (COVID-19) is caused by the beta-coronavirus severe acute respiratory syndrome coronavirus-2 (SARS-CoV-2) [1]. The leading cause of death in patients with COVID-19 is hypoxic respiratory failure secondary to acute respiratory distress syndrome (ARDS). COVID-19 presents with a spectrum of clinical phenotypes, with most patients exhibiting either mild or moderate symptoms. However, approximately 15% of patients progress to more severe disease necessitating hospitalization and cardiopulmonary support [2, 3]. Current epidemiological data suggests that COVID-19 has a mortality rate several times greater than that of seasonal influenza [4]. Additionally, elderly patients and patients with underlying comorbidities such as cardiovascular disease, diabetes mellitus, chronic lung disease, chronic kidney disease, obesity, and cancer have a higher risk of COVID-19 complications and an increased mortality rate compared with infected young, healthy adults [5]. Circulating inflammatory and vascular markers have been associated with more severe SARS-CoV-2 infections (troponin, D-dimer, lymphocyte counts, and some inflammatory cytokines), suggesting a mechanistic link between vascular and immune dysfunction in COVID-19 [6].

Multi-organ damage in COVID-19 is related to unchecked inflammation and direct viral-induced organ and cell dysfunction. SARS-CoV-2 infects the host through interactions with the angiotensin-converting enzyme 2 (ACE2) receptor [7]. The ACE2 receptor is expressed in the lung, heart, kidney, and intestine. ACE2 receptors are also expressed by endothelial cells (EC). Whether vascular derangements in COVID-19 are due to viral infection of EC or immune-related pathology (or some combination of the two) in response to the virus remains unknown. To date, EC have been largely overlooked as a therapeutic target in COVID-19; emerging evidence suggests that these cells contribute to the initiation and propagation of ARDS in COVID-19 by altering vascular integrity, promoting micro- and macrovascular thrombosis, and inducing vascular inflammation [8]. A mechanistic understanding of the direct and indirect SARS-CoV-2 effects on the vasculature is critical and will help to clarify vascular-immune interactions that underlie the pathobiology of COVID-19.

As our knowledge of COVID-19 has evolved, it is clear that vascular and thrombotic complications are common in COVID-19 [9]. A recent study found evidence of direct viral-mediated dysfunction of the vascular endothelium in a series of patients suffering from severe COVID-19 [10]. Additionally, in a small cohort of COVID-19 patients, Rovas and colleagues performed intravital microscopy to quantify vascular density and GAC properties in sublingual microvessels [11]. They found that COVID-19 patients had striking reductions in microvascular density and had evidence of GAC damage providing direct clinical evidence of vascular dysfunction. The endothelial GAC is comprised of proteoglycans, glycosaminoglycan (GAG) chains, and glycoproteins [12]. Syndecan-1 (SDC1), a canonical proteoglycan, helps maintain vascular integrity and regulates endothelial responses. GAG chains that bind to proteoglycans include chondroitin sulfate and heparan sulfate, some of which have been implicated in SARS-CoV-2 infectivity [13]. In contrast to the GAGs described above, hyaluronan (Hyal) is a linear, neutral molecule that interacts with cell-membrane CD44 and can form complexes with other GAGs, complexes that together stabilize the GAC. In sepsis, the GAC can be actively degraded by enzymes including metalloproteinases, heparanase, and hyaluronidase [14]. Immune-mediated GAC degradation increases vascular permeability, microvascular thrombosis, and leukocyte recruitment [15]. Observational studies in sepsis populations have found an association between circulating levels of GAC degradation products and end-organ dysfunction and mortality [16].

Similar to the endothelial GAC disruption in sepsis-mediated ARDS, we hypothesized that endothelial GAC disruption is associated with vascular dysfunction in SARS-CoV-2 infections and precedes the genesis of ARDS in COVID-19. The objective of this study was to evaluate if GAC components and inflammatory biomarkers were elevated in hospitalized COVID-19 patients compared with healthy controls (HC) and if these markers correlated with disease severity. We found that Hyal and SDC1 were increased in COVID-19 patients and these markers correlated with disease severity. This study builds on prior studies and further reveals an important intersection between GAC remodeling and SARS-CoV-2 infections, an intersection which could have diagnostic and therapeutic implications and furthers our understanding of the pathobiology of COVID-19.

## Materials and Methods

### Enrolled Patients

This study was approved by the Research and Development and Institutional Review Board committees at the LTC Charles S. Kettles VA Medical Center (Ann Arbor, MI). Informed consent was waived for the study. We analyzed plasma samples obtained at hospital admission for 12 symptomatic COVID-19 reverse transcription polymerase chain reaction (RT-PCR)–confirmed cases that presented to the LTC Charles S. Kettles VA Medical Center. Blood samples from patients with COVID-19 were drawn for clinical purposes and obtained from the hospital pathology laboratory following completion of clinical analysis, and were de-identified prior to analysis by the study laboratory team. Patient samples were stored at 4 °C for up to 48 h prior to collection by the study team, then were frozen at − 80 °C until analysis. Blood plasma was also collected from 7 healthy individuals (SARS-CoV-2 negative) who were used as controls. Control samples were acquired at the University of Illinois at Chicago prior to the SARS-CoV-2 global outbreak from participants residing in the Greater Chicagoland area after providing written informed consent. All control samples were acquired after a standardized period (8–12 h) of overnight fasting.

### Sample Processing

The concentration of SDC1 in plasma samples was determined in duplicates via solid-phase sandwich Human sCD138 (SDC1) ELISA Kit (Diaclone, Cat.# 950.640.192) following the manufacturer’s protocol, and calculated by extrapolating OD values against CD138 standard concentrations using the standard curve. The concentration of Hyal in plasma samples was determined in duplicates via Quantikine® Colorimetric Sandwich ELISA Kit (R&D Systems, Cat.# DHYALO) following the manufacturer’s protocol, and calculated by extrapolating OD values against Hyaluronan standard concentrations using four parameter logistic (4-PL) curve fit. Plasma cytokines were profiled using the MILLIPLEX MAP Human High Sensitivity T Cell Panel Premixed 13-plex (Millipore, Cat.# HSTCMAG28PMX13BK) according to the manufacturer’s instructions. Briefly, each plate was blocked with wash buffer for 10 min before use. The mixed beads were dispensed into each well and washed twice. The standard curve was generated by reconstituting the high-sensitivity human cytokine standard, per the manufacturer’s protocol, with serial 1:5 dilutions for a working concentration range of 0.13–400 pg/mL. The Millipore kits also include quality control (QC) material to confirm the accuracy of the assay. Each analyte in the QC sample was intended to fall within a designated range, and if these values deviated from this range, the data was considered invalid. The samples and standards were incubated with the mixed beads overnight at 4 °C while shaking. The beads were washed and then incubated with a detection antibody at room temperature for 1 h and with streptavidin for an additional 30 min. The beads were washed twice and resuspended in Luminex MagPix® drive fluid, and the plate was subsequently analyzed on the Luminex MagPix® plate reader. The MFI was then compared to the standard curve, as previously described, to calculate the cytokine concentration in picograms per milliliter. Each standard curve was then individually analyzed for outliers and adjusted as necessary to achieve linearity (R2 ≥ 0.8). All ELISAs and Luminex assays were performed by operators blinded to subject clinical characteristics including the COVID-19 status.

### Statistical Analysis

Due to non-normality, data are presented as median (interquartile range), with between-group comparison using Mann–Whitney testing, and *p* value < 0.05 was considered significant. The analyses were performed and depicted using GraphPad Prism (GraphPad Software, La Jolla, CA). Bivariate correlations were determined by use of the Pearson correlation coefficient.

## Results

### Patient Cohort and Clinical Characteristics

We prospectively enrolled 12 veteran patients hospitalized with COVID-19 between May 14 and August 10, 2020. All patients had SARS-CoV-2 confirmed by RT-PCR of the nasal swab or tracheal aspirate. The characteristics of this patient population and healthy controls are presented in Table 1. Hospitalized patients with COVID-19 were male older adults with a high prevalence of medical comorbidities such as heart failure, obesity, diabetes, and kidney disease.

**Table 1:** Subject characteristics

Variable	COVID-19	Control
*n*	12	7
Race (% Caucasian)	67	71
Age, years	71 ± 10	26 ± 4*
BMI, kg/m^2^	31.3 ± 6.1	21.4 ± 2.1*
Heart failure (%)	33	0
Hypertension (%)	92	0
COPD (%)	33	0
CKD (%)	67	0
T2DM (%)	50	0
Cancer (%)	8	0

Data represent mean ± SD

*BMI* body mass index

^*^Significant difference between COVID-19 and control (*p* < 0.05)

### COVID-19 Associated with Elevated Inflammatory Cytokines

**Fig. 1:**
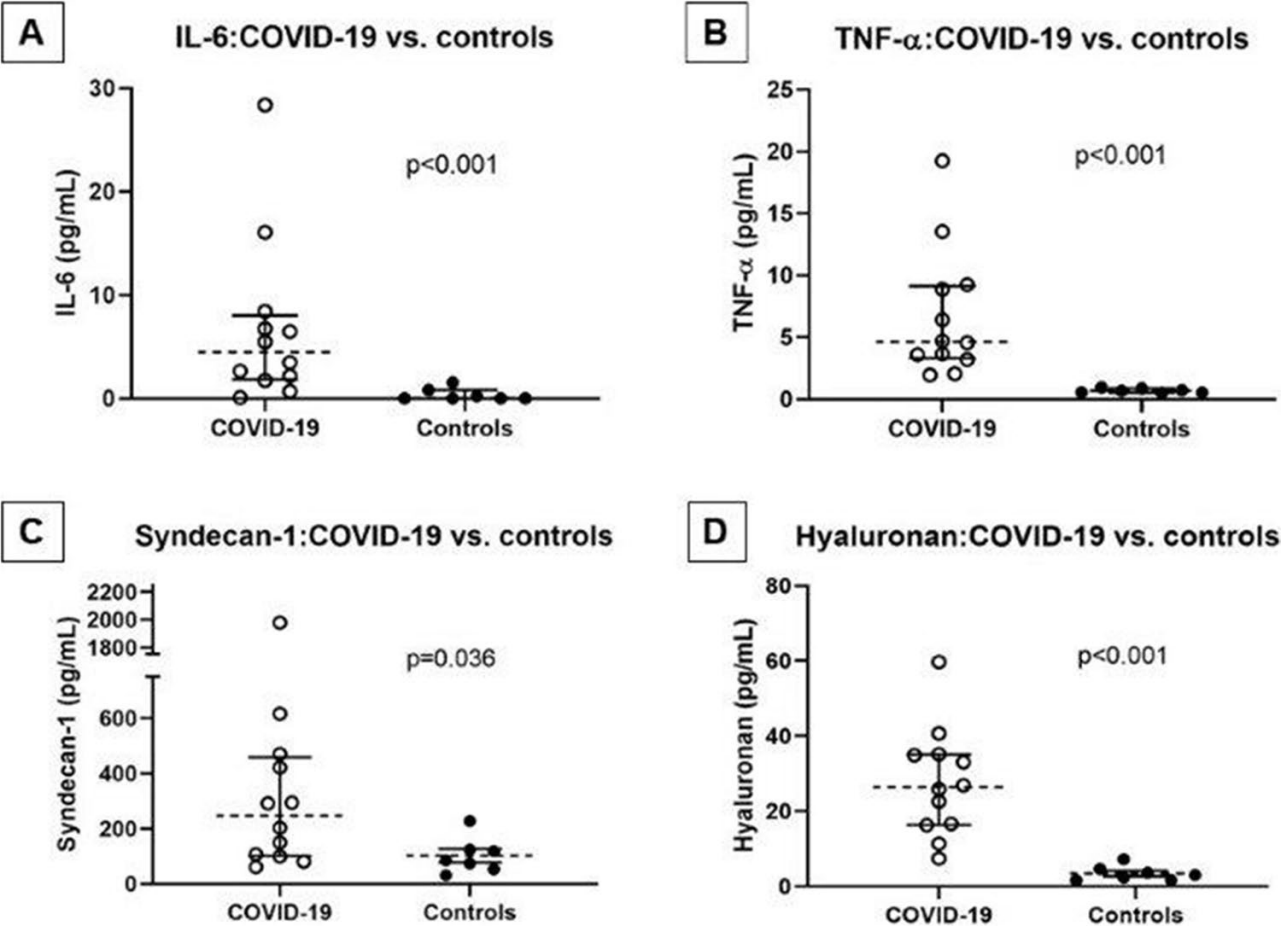
Inflammatory biomarkers and glycocalyx components in COVID-19 vs. controls. Abbreviations: IL-6: interleukin-6; TNFα: tumor necrosis factor alpha. Statistical comparisons with Mann–Whitney testing; line: median; whiskers: interquartile range

Numerous studies have found that patients hospitalized with COVID-19 have elevated inflammatory markers [17, 18]. To characterize our patient cohort and to put it into context with other observational studies, we ran Luminex assays on plasma from our COVID-19 cohort. In agreement with other studies, we found that IL-6 and TNFα were elevated in the COVID-19 cohort compared to the control subjects [median (IQR), all units picograms per milliliter; IL-6: 4.65 (3.32–9.16) vs 0.69 (0.55–0.89), *p* < 0.001; TNFα: 4.49 (1.87–8.03) vs 0.04 (0.04–0.84), *p* < 0.001] (Fig. 1a, b). IL-6 and TNFα are classical inflammatory markers, and their upregulation is indicative of a robust immune response; however, sustained elevations of inflammatory mediators suggest a hyperactive immune response which ultimately can be detrimental to the host.

### COVID-19 Associated with Elevated GAC Degradation Products

Hyperactivation of the immune system is a common manifestation of SARS-CoV-2 infections. In parallel, SARS-CoV-2 can cause epithelial and endothelial damage. In addition to elevation in inflammatory cytokines, we found that GAC components, SDC1 and Hyal, were elevated on admission compared to controls [median (IQR), all units picograms per milliliter; SDC1: 247.37 (101.43–458.26) vs 84.8 (52.88–123.59), *p* = 0.036; Hyal: 26.41 (16.4–35.1) vs 3.01 (1.66–4.61), *p* < 0.001] (Fig. 1c, d).

### Relationship Between Immune Activation and Markers of GAC Disruption in COVID-19

Correlations between markers of immune activation and the GAC biomarkers SDC1 and Hyal in the COVID-19 cohort are presented in Table 2. Importantly, although there were significant associations between markers of inflammation and GAC disruption (IL-6 and IL-8 with both Hyal and SDC1), there was also heterogeneity (for example, TNFα with Hyal but not SDC1) suggesting specific interactions between different arms of the immune response and GAC degradation.

**Table 2:** Correlation matrix of immune activation markers and GAC biomarkers. Pearson correlation coefficient (*r*) and *p* values are reported for each bivariate correlation. Significant (*p* < 0.05) correlations are indicated in bold text

	**Syndecan-1**	**Hyaluronan**	**IL-10**	**IL-1b**	**IL-2**	**IL-4**
**Syndecan-1**	1					
**Hyaluronan**	0.42 0.0734	1				
**IL-10**	0.1552 0.5258	0.5062 0.027	1			
**IL-1b**	0.4303 0.0659	0.2176 0.3708	0.5437 0.0161	1		
**IL-2**	− 0.1218 0.6194	− 0.206 0.3976	0.1353 0.5809	0.5066 0.0269	1	
**IL-4**	0.7334 0.0004	0.1318 0.5908	0.0648 0.7923	0.5344 0.0184	0.0115 0.9626	1
**IL-5**	0.8741 < 0.001	0.2012 0.4089	0.081 0.7418	0.5839 0.0087	0.0029 0.9907	0.9468 <0.001
**IL-6**	0.7896 0.0001	0.5854 0.0085	0.2543 0.2934	0.5895 0.0079	− 0.1103 0.653	0.7829 0.0001
**IL-8**	0.8663 < 0.001	0.6374 0.0033	0.4231 0.0711	0.5795 0.0093	− 0.134 0.5843	0.7954 < 0.001
**TNFα**	0.1281 0.6012	0.8601 < 0.001	0.4799 0.0376	0.2117 0.3844	− 0.1937 0.4268	0.0163 0.9472

## Discussion

In this study, we found that inflammatory biomarkers (IL-6 and TNFα) and GAC markers (Hyal and SDC1) were elevated in hospitalized COVID-19 patients compared with HC. Additionally, we found that not all circulating immune markers are associated with GAC remodeling, further reinforcing the notion that vascular and immune axes are regulated specifically and are dysfunctional in severe SARS-CoV-2 infections.

Although a majority of COVID-19 patients either are asymptomatic or have only mild symptoms, a subset of COVID-19 patients develops severe respiratory symptoms and ARDS. The reasons for the heterogeneous clinical presentation and the pathologic mechanisms that underlie respiratory failure still are not fully understood. Studies over the last year have suggested several mechanisms linked to vascular function that may contribute to the progression of COVID-19 [19]. First, to enter cells, SARS-CoV-2 binds to the ACE2 receptor which impairs ACE2 activity. Impaired ACE2 activity leads to elevated and sustained angiotensin vascular dysfunction and also indirectly activates the kallikrein–bradykinin pathway which leads to in increased vascular permeability. Second, activated immune cells are recruited to pulmonary vasculature and produce reactive oxygen species (ROS), inflammatory cytokines, and other mediators which can disrupt the pulmonary vascular barrier. Finally, SARS-CoV-2 can have direct and indirect effects on pulmonary endothelial cells themselves. SARS-CoV-2 can trigger endothelial inflammation and the production and release of inflammatory cytokines such as IL-1β, TNFα, and IL-6. These inflammatory cytokines can activate enzymes that degrade the endothelial GAC, resulting in endothelial dysfunction, a prothrombotic endothelial phenotype, and cell death, all of which are hallmarks of ARDS and present in severe COVID-19. In addition to the immune-mediated consequences described above, COVID-19 is associated with a high incidence of micro- and macrovascular thrombosis [20]. Because of these associations, several randomized clinical trials have explored the clinical effects of therapeutic anticoagulation compared with thromboprophylaxis in COVID-19. Because of the possible immune-modulating effects (in addition to its anticoagulant effects), heparins have been an attractive therapeutic for COVID-19. In support of this, two recent large clinical trials in noncritically ill COVID-19 patients suggested that an initial strategy of therapeutic-dose anticoagulation with heparin increased the chance of survival to hospital discharge with reduced use of cardiovascular or respiratory organ support as compared with usual-care thromboprophylaxis [21, 22].

Our findings are in agreement and build on the findings of several recent studies. From a mechanistic standpoint, Mycroft-West and colleagues found that heparin, an anticoagulant molecularly similar to the GAC component heparan sulfate, binds the Spike (S1) protein receptor binding domain and inhibits SARS-CoV-2 viral invasion by approximately 70% [13]. This provocative study not only confirmed that components of the GAC are important for direct viral invasion of endothelial cells but also suggested that treatment with heparin could have beneficial effects in COVID-19, independent of its anticoagulant properties. In a related study, Buijsers and colleagues found that heparanase activity and heparan sulfate levels were elevated in hospitalized COVID-19 patients compared with those in HC [23]. In mechanically ventilated COVID-19 patients, Stahl and colleagues found evidence of endothelial GAC disruption with elevated Tie2 and SDC1 compared with HC [24]. Our observations from a more general hospitalized COVID-19 cohort add to the evidence that GAC remodeling is associated with and potentially a pathologic driver of endothelial dysfunction and ARDS in COVID-19. Additionally, the strong correlations between specific (not all) circulating cytokines and GAC markers suggest that immune–GAC interactions are likely more specific than simple associations between inflammation and vascular dysfunction.

There are several limitations of this study. First, because of restrictions on patient interactions, the samples collected were not always within 6 h of presentation. Because of the natural immune arc to SARS-CoV-2 infections, this undoubtedly introduced heterogeneity into the study. Second, this study was not designed nor powered to evaluate the performance of GAC markers in predicting clinical outcomes; however, we believe our findings build on a growing body of evidence regarding the importance of GAC in vascular dysfunction in COVID-19 and suggest hypotheses that warrant further validation in larger, prospective studies. Finally, even though consecutive patients admitted to our institution were enrolled in this study, because of the limited hospitalized non-COVID-19 patients during the period of this study, we lack a pure non-COVID-19 control cohort that more closely matches our COVID-19 cohort.

In summary, circulating GAC degradation products were elevated in hospitalized COVID-19 patients suggesting that vascular injury and GAC remodeling contribute to the morbidity and mortality in SARS-CoV-2 infections. The parallel assessment of GAC markers with inflammatory cytokines may help to identify patients at risk of COVID-19 complications, and further understanding of the bidirectional relationship between specific immune and GAC responses in COVID-19 is certain to advance our understanding of how inflammation and vascular dysfunction coexist. Larger prospective clinical studies with defined GAC and inflammatory markers are required to determine the principal effects of SARS-CοV-2 on vascular and immune endothelial function and the underlying pathophysiological mechanisms that unite the two. However, these findings further support the notion that vascular dysfunction occurs in tandem with immune dysregulation and could be leveraged to identify patients who are at high risk and also could identify novel therapeutic approaches towards COVID-19 and other immune-vascular diseases.

## Data Availability

Formal requests to access the dataset need to be sent to the corresponding authors (Hummel, Goonewardena).
